# Lactate to albumin ratio as a determinant of intensive care unit admission and mortality in hospitalized patients with community-acquired pneumonia

**DOI:** 10.1186/s12890-025-03698-7

**Published:** 2025-05-09

**Authors:** Pervin Hancı, Esra Temel, Furkan Bilir, Bilkay Serez Kaya

**Affiliations:** 1 Department of Pulmonary Medicine and Division of Intensive Care, Trakya University Faculty of Medicine, Trakya University Medical Research and Application Centre, Edirne, TR-22030 Turkey; 2 Department of Pulmonology, Trakya University Faculty of Medicine, Edirne, Türkiye

**Keywords:** Pneumonia, Intensive care units, Hospitalization, Mortality, Lactate, Albumin

## Abstract

**Objective:**

Previous studies have shown that the lactate/albumin ratio (LAR) can be a prognostic biomarker in intensive care unit (ICU) patients. However, the usefulness of LAR in predicting mortality and guiding intensive care unit admission in hospitalized patients due to community-acquired pneumonia (CAP) remains unclear. This study aims to evaluate the predictive value of the LAR compared to Pneumonia Severity Index (PSI), Confusion, urea, respiratory rate, blood pressure, 65 years or older (CURB-65), and quick-Sequential Organ Failure Assessment (q-SOFA) scores in determining the need for ICU admission and mortality among hospitalized patients with CAP.

**Methods:**

Adult patients diagnosed and hospitalized with community-acquired pneumonia between July 2021 and July 2023 were included. Patients’ demographics, comorbidities, disease severity scores, laboratory findings at the admission and outcomes were recorded. Patients were grouped and compared according to admission place (ward or ICU).

**Results:**

PSI, CURB-65, q-SOFA scores, and LAR were higher in ICU patients than in those admitted to the ward. Regarding the ICU admission, the AUC values for PSI, CURB-65, q-SOFA and LAR were 0.794 (95% CI: 0.737–0.843) (*p* < 0.001), 0.825 (95% CI: 0.771–0.870) (*p* < 0.001), 0.755 (0.690–0.813) (*p* < 0.001), and 0.749 (95% CI: 0.689–0.802) (*p* < 0.001), respectively. Regarding the mortality, the AUC values for PSI, CURB-65, q-SOFA, and LAR were 0.722 (95% CI: 0.661–0.778) (*p* < 0.001), 0.743 (95% CI: 0.683–0.797) (*p* < 0.001), 0.645 (0.575–0.711) (p: 0.02), 0.761 (95% CI: 0.702–0.814) (*p* < 0.001), respectively. There wasn’t any difference detected in pairwise comparisons of ROC curves.

**Conclusion:**

In this study, LAR was found to be a good predictor of ICU admissions and mortality in hospitalized patients with CAP and was non-inferior to PSI, CURB-65, or q-SOFA scores.

**Supplementary Information:**

The online version contains supplementary material available at 10.1186/s12890-025-03698-7.

## Introduction

Community-acquired pneumonia (CAP) is a lower respiratory tract infection that impacts individuals of all ages. CAP is a significant cause of hospitalization and mortality, especially in the elderly and immune-compromised patients [1]. The mortality rate for hospitalized patients with CAP ranges from 5 to 10%. However, in cases where patients need to be admitted to the intensive care unit (ICU), the mortality rate is significantly higher. Those admitted to the ICU are likely to have severe or life-threatening infections that require advanced medical interventions, such as mechanical ventilation and hemodynamic support. The mortality rate for ICU patients with CAP can range from 30 to 54% [2, 3].

Early diagnosis and treatment approaches will significantly affect the prognosis. Although scoring systems such as CURB-65 (Confusion, urea, respiratory rate, blood pressure, 65 years or older) and PSI (pneumonia severity index), and more recently SOFA (Sequential Organ Failure Assessment) and q-SOFA (quick SOFA) have been very useful in patient follow-up and management and defining mortality risk for CAP, there is still a need for biomarkers that will provide practicality in showing prognosis [4–7]. With these accessible biomarkers with high sensitivity and specificity, risky conditions could be recognized in the early period, and mortality and morbidity in cases with CAP could be significantly reduced with appropriate treatment approaches.

Arterial lactate level is known as a measure of cellular oxygenation, and a high lactate level is considered an indicator of tissue hypoxia [8], which was found to be one of the poor prognostic indicators in cases of pneumonia [9]. The prognostic value of lactate levels is important in clinical practice in the follow-up of patients and in determining treatment strategies. High lactate levels may indicate the need for intensive care, mechanical ventilation, and other serious complications. Therefore, regularly monitoring lactate levels in pneumonia patients could be critical for early intervention and a more effective treatment plan.

Albumin plays a critical role as a key plasma protein, responsible for regulating fluid balance, maintaining oncotic pressure, supporting immune function, and serving as a marker of inflammation severity as a negative acute phase protein [10]. Several studies have highlighted the association between low blood albumin concentration and poor prognosis in pneumonia, sepsis, and cancer [11–13]. The relationship between hypoalbuminemia and pneumonia could be explained by compromised immune responses, impaired tissue repair, and increased susceptibility to secondary infections.

Since lactate and albumin independently predict prognosis in pneumonia, the lactate-to-albumin ratio (LAR) may provide a better prognostic performance. It has been proven in many studies that the lactate/albumin ratio determines prognosis in critically ill patients [14–18]. However, its power to predict ICU admission and mortality in hospitalized CAP patients remains unclear.

This study aimed to evaluate the predictive value of the LAR for ICU admission and 28-day mortality in hospitalized patients with CAP.

## Method

This retrospective cohort study was conducted at Trakya University Faculty of Medicine Hospital. This study was conducted in accordance with the principles of the Declaration of Helsinki and has been approved by the Trakya University Faculty of Medicine Noninterventional Scientific Research Ethics Committee with the reference number TUTF-GOBAEK 2023/421. (Clinical trial number: not applicable) Upon admission to the clinic, patients or their legally authorized relatives provided written informed consent for the processing and publishing of their medical records (with names disclosed) for scientific purposes in accordance with the clinic’s regulatory procedures.

### Patients

The study enrolled patients who were diagnosed and hospitalized with CAP between July 2021 and July 2023 and were at least 18 years old. The inclusion criteria for CAP included the presence of lower respiratory tract infection symptoms such as cough, sputum, shortness of breath, chest pain, fever or hypothermia, and confusion. Additionally, new infiltrates on a chest radiograph should be identified, and there should be no alternative diagnosis present. Patients who did not meet the inclusion criteria hadn’t required hospitalization, had a history of hospitalization for two days or more within the preceding 90 days, were residents in a nursing home, were using infusion therapy at home, had received chronic dialysis within 30 days, had been taking wound care or history of infection with a multidrug-resistant pathogen in a family member, had laboratory-confirmed coronavirus disease-2019 (COVID-19) were excluded from the study.

### Data

Medical records of the patients screened, and patients’ demographic data (age, gender), comorbidities, white blood cell count (WBC), serum albumin and lactate levels, PSI, CURB-65 and q-SOFA scores at admission; admission place [ward or ICU], and outcomes (ICU and hospital length of stay, hospital mortality) were recorded. The LAR was calculated.

### Statistical analyses

IBM SPSS software version 26.0 (IBM Corporation, Armonk, NY) was used for statistical analyses. Descriptive analyses were presented as numbers (percentages) for categorical variables or median [25th-75th percentile] for numerical variables. Patients were grouped according to the admission place (ward and ICU). Age, PSI, CURB-65 scores, q-SOFA, WBC, lactate and serum albumin values, LAR, and length of stay in the hospital were compared with the Mann-Whitney U or Student T-tests according to normal distribution characteristics. Categorical data such as gender, comorbidities, need for mechanical ventilation during hospitalization, need for vasopressor support, and hospital mortality were evaluated by Chi-square analysis or Fisher exact test. Receiver operator curve (ROC) analysis was performed to evaluate the predictive prognostic efficacies of the PSI, CURB-65, q-SOFA and LAR for ICU admission and the hospital mortality of all patients. Optimal cut-off values and area under the curves (AUC) were calculated using MedCalc software (MedCalc Software Ltd, Ostend, Belgium). The method described by DeLong et al. was utilized to compare ROC curves [19]. A 5% type-I error level will be used to infer statistical significance.

## Results

**Fig. 1:**
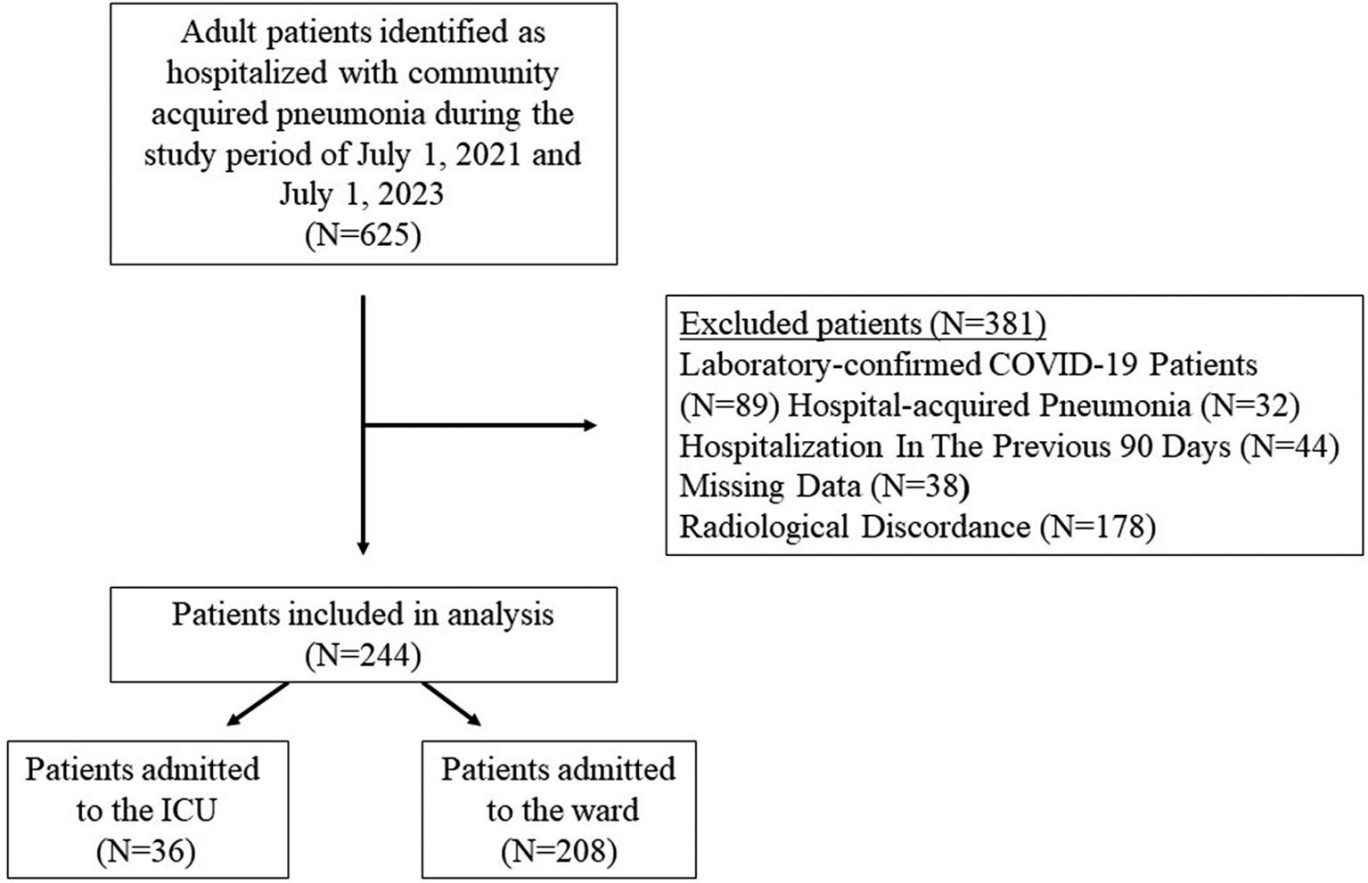
Flow chart

The records of 625 patients hospitalized due to CAP during the study period were screened from the hospital database based on the International Classification of Diseases (ICD-9) Code. 381 patients were excluded for the following reasons shown in Fig. 1. Of the 244 patients who fulfilled the inclusion criteria, 36 were admitted to the ICU and 208 to the ward.

Table 1 presents the baseline characteristics, disease severity score points, and outcomes of 244 patients based on their admission place. The median age of patients was 72 [65–79]. 68% of the patients were men, and hypertension was the leading comorbidity (57.4%). The median PSI score was 108 [91–135], the median CURB-65 score was 2 [1–2], and the median q-SOFA was 1 [0–1]. The median lactate-to-albumin ratio was 0.40 [0.28–0.61]. In-hospital mortality was 11% (*n* = 22) overall. Nineteen patients (7.7%) were intubated and mechanically ventilated at the ICU admission.

**Table 1:** Baseline characteristics and outcomes of patients according to admission place

	All (*n* = 244)	Ward (*n* = 208)	ICU (*n* = 36)	*p*
Age, year	72[65–79]	72 [65–79]	71 [62–81]	0.67
Gender (Male)*	166 (68)	139 (66.8)	27 (75)	0.32
History of smoking	120 (49.1)	107 (51.4)	13 (36.1)	0.58
Any Comorbidity*	233 (95.5)	197 (94.7)	36 (100)	0.19
Hypertension*	140 (57.4)	119 (57.2)	21 (58.3)	0.90
Malignity*	82 (33.6)	70 (33.7)	12 (33.3)	0.97
Coronary arterial disease*	72 (29.5)	61(29.3)	11 (30.6)	0.88
Diabetes mellitus*	71 (29.1)	63 (30.3)	8 (22)	0.32
COPD*	64 (26.2)	51 (24.5)	13 (36.1)	0.14
Neurological disease*	45 (18.4)	32 (15.3)	13 (36.1)	**0.003**
Heart Failure*	31 (12.7)	25 (12)	6 (16.3)	0.29
Chronic renal disease*	29 (11.9)	25 (12)	4 (11.1)	0.56
Other comorbidities*	18 (7.3)	18 (8.6)	0 (0)	0.08
PSI score	108 [91–135]	106 [88–129]	141[116–178]	**< 0.001**
CURB-65	2 [1–2]	1 [1–2]	3 [2–4]	**0.002**
q-SOFA	1 [0–1]	1 [0–1]	1 [1–2]	**< 0.001**
WBC (10^9^/L)	11.6 [8.1–15.8]	11.6 [8.2–15.6]	11.6 [7.5–17.1]	0.62
Lactate (mmol/L)	1.4 [1.0-2.1]	1.4 [1.0-1.9]	2.2 [1.4–3.2]	**< 0.001**
Albumin (g/dL)	3.6 [3.1–3.9]	3.6 [3.2-4.0]	3.1 [2.6–3.6]	**< 0.001**
Lactate/albumin ratio	0.40 [0.28–0.61]	0.36 [0.27–0.54]	0.8 [0.4–1.1]	**< 0.001**
Hospital LOS (day)	10 [6–18)	10 [6–16]	19 [8–25]	0.92
Hospital Mortality*	27 (11.1)	9 (4.3)	18 (50)	**< 0.001**

Definition of Abbreviations: COPD: Chronic obstructive pulmonary disease, PSI: Pneumonia severity index, CURB-65: Confusion, blood urea nitrogen, respiratory rate, blood pressure, age > 65, qSOFA: Quick sequential organ failure assessment, WBC: White blood cell, IMV: Invasive mechanical ventilation, LOS: Length of stay. Data expressed in median [interquartile range], and *: n (%)

Patients were grouped and compared according to admission place (Table 1). Groups were not different in terms of age, gender, comorbidities except for neurological disease, WBC, and hospital length of stay. Neurological diseases were more common in ICU patients (36.1%) than in ward patients (15.3%) (*p* = 0.003). PSI [151 ± 45.3 vs. 106.7 ± 30.7, t:5.7 (95% CI: 29–60)(*p* < 0.001)], CURB-65 [2.89 ± 1.2 vs. 1.42 ± 0.8, t:7, (95% CI: 1.0-1.8)(*p* < 0.001)], q-SOFA (1 [1–2] vs. 1[0–1], z: -5.2, *p* < 0.001) and LAR [0.91 ± 0.71 vs. 0.44 ± 0.26, t:3.8 (95% CI 0.22–0.71)(*p* < 0.001)] were higher in ICU patients than in ward patients. In-hospital mortality was lower in ward patients (4.3%) compared to ICU patients (50%) (*p* < 0.001). PSI, CURB-65 scores, q-SOFA, and LAR were higher in nonsurvivors than in survivors (Table 2).

**Table 2:** Pneumonia severity indices according to the mortality

	Survivor (*n* = 217)	Nonsurvivor (*n* = 27)	*p*
PSI	107 [89–132]	136 [108–139]	< 0.001
CURB-65	1 [1–2]	3 [2–4]	< 0.001
q-SOFA	1 [0–1]	1 [0–2]	0.009
Lactate/albumin ratio	0.36 [0.27–0.56]	0.76 [0.45–1.22]	< 0.001

Definition of Abbreviations: PSI: Pneumonia severity index, CURB-65: Confusion- blood urea nitrogen-respiratory rate-blood pressure- age > 65, qSOFA: Quick-Sequential Organ Failure Assessment

**Fig. 2:**
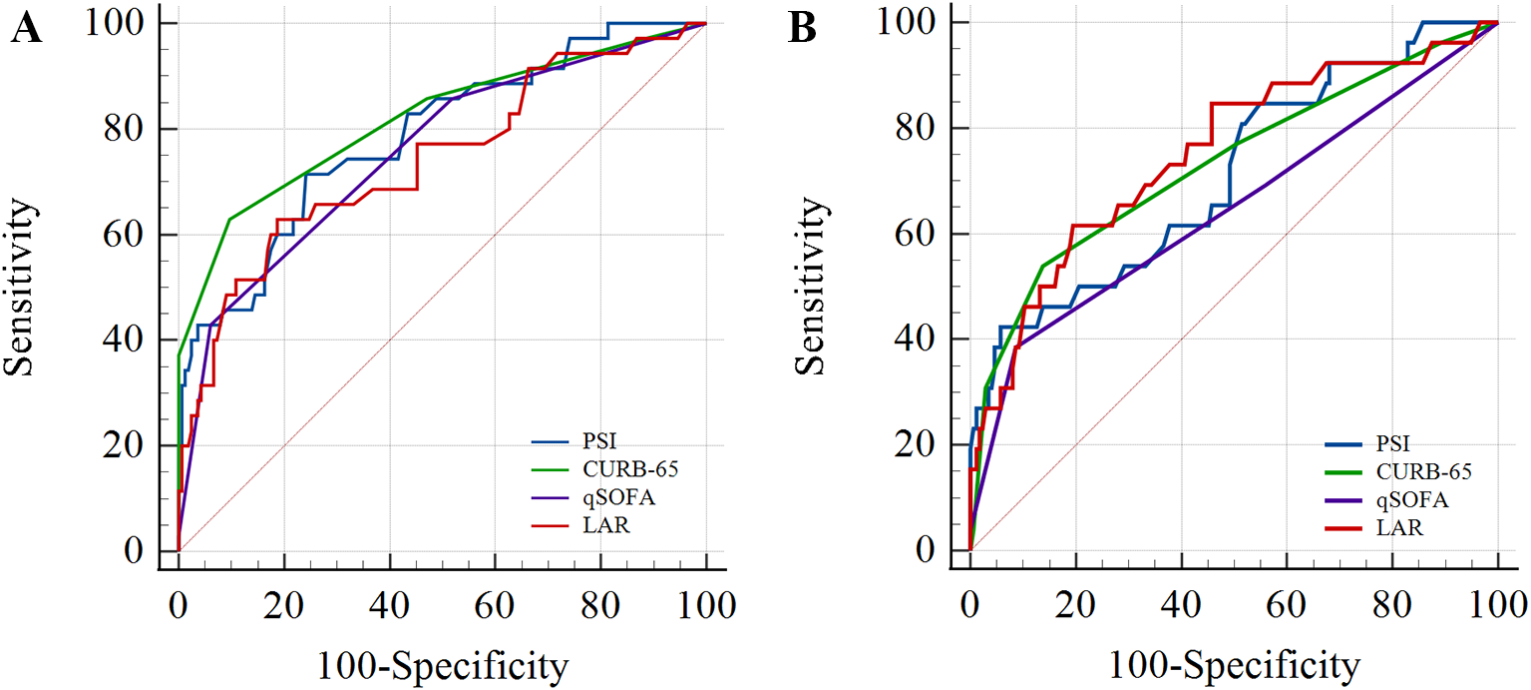
ROC curve analysis regarding the ICU admission (**A**) and hospital mortality (**B**). Definition of Abbreviations: PSI: Pneumonia severity index, CURB-65: Confusion- blood urea nitrogen-respiratory rate-blood pressure- age > 65, q-SOFA: Quick sequential organ failure assessment, LAR: Lactate–albumin ratio

ROC curves were used to investigate and compare the predictive function between PSI, CURB-65, q-SOFA and LAR for ICU admission and mortality (Fig. 2). Regarding the ICU admission, the AUC values for PSI, CURB-65, q-SOFA and LAR were 0.794 (95% CI: 0.737–0.843) (*p* < 0.001), 0.825 (95% CI: 0.771–0.870) (*p* < 0.001), 0.755 (0.690–0.813) (*p* < 0.001), and 0.749 (95% CI: 0.689–0.802) (*p* < 0.001), respectively (Fig. 2A; Table 3). The optimal cut-off values and corresponding sensitivity and specificity of PSI, CURB-65 scores, q-SOFA, and LAR regarding ICU admission are shown in Table 3. The PSI score > 129 had the highest sensitivity (69.4%), while the CURB-65 score > 2 had the highest specificity (91.8%) for ICU admission. Regarding the mortality, the AUC values for PSI, CURB-65, q-SOFA, and LAR were 0.722 (95% CI: 0.661–0.778) (*p* < 0.001), 0.743 (95% CI: 0.683–0.797) (*p* < 0.001), 0.645 (0.575–0.711) (p: 0.02), 0.761 (95% CI: 0.702–0.814) (*p* < 0.001), respectively (Fig. 2B) (Table 4). The optimal cut-off values and corresponding sensitivity and specificity of PSI, CURB-65 scores, q-SOFA, and LAR regarding mortality are shown in Table 4. The LAR > 0.6 displayed the highest sensitivity (61.5%), while the CURB-65 had the greatest specificity (88.4%). There was no difference in pairwise comparisons of the ROC curves of the PSI, CURB-65, q-SOFA, and LAR in predicting ICU admission (Supplementary Table 1) or mortality (Supplementary Table 2).

**Table 3:** The AUC values and corresponding confidence intervals for the PSI, CURB-65, q-SOFA scores, and Laktad/albumin ratio; the optimal cut-off values and corresponding sensitivity and specificity for each scoring system regarding intensive care unit admission among hospitalized patients with community-acquired pneumonia

Score	AUC (95% CI)	*p*	Cut-off	Sensitivity (%)	Specificity (%)
PSI	0.794 (0.737–0.843)	< 0.001	129	69.4	76.7
CURB-65	0.825 (0.771–0.870)	< 0.001	2	63.9	91.8
q-SOFA	0.755 (0.690–0.813)	< 0.001	1	42.9	84.0
Lactate/albumin	0.749 (0.689–0.802)	< 0.001	0.60	62.9	81.2

Definition of Abbreviations: PSI: Pneumonia severity index, CURB-65: Confusion- blood urea nitrogen-respiratory rate-blood pressure- age > 65, q-SOFA: Quick sequential organ failure assessment, CI: Confidence intervals

**Table 4:** The AUC values and corresponding confidence intervals for the PSI, CURB-65, q-SOFA scores, and Laktad/albumin ratio; the optimal cut-off values and corresponding sensitivity and specificity for each scoring system regarding mortality among hospitalized patients with community-acquired pneumonia

Score	AUC (95% CI)	*p*	Cut-off	Sensitivity (%)	Specificity (%)
PSI	0.722(0.661–0.778)	< 0.001	129	48.1	74.4
CURB-65	0.743 (0.683–0.797)	< 0.001	2	55.5	88.4
q-SOFA	0.645 (0.575–0.711)	0.02	1	38.4	81.4
Lactate/albumin	0.761 (0.702–0.814)	< 0.001	0.63	61.5	81.1

Definition of Abbreviations: PSI: Pneumonia severity index, CURB-65: Confusion- blood urea nitrogen-respiratory rate-blood pressure- age > 65, q-SOFA: Quick sequential organ failure assessment, CI: confidence intervals

## Discussion

This study demonstrated that the LAR is a useful biomarker in predicting ICU admission and mortality in patients hospitalized with CAP. When compared with traditional clinical prediction tools such as PSI, CURB-65, and q-SOFA, LAR showed comparable predictive value for both ICU admission and in-hospital mortality. Notably, there was no statistically significant difference in ROC curve comparisons between these four parameters, supporting the non-inferiority of LAR.

CAP remains a critical health concern, characterized by high morbidity and mortality. Numerous predictive models and severity scores have been developed to guide clinical decisions regarding hospitalization and ICU admission and assess the mortality risk of CAP patients. The Clinical Practice Guidelines of the American Thoracic Society and Infectious Diseases Society recommends using PSI and CURB-65 scoring systems to determine the need for hospitalization [20]. Furthermore, extensive research has confirmed the effectiveness of these scoring systems in predicting the necessity for intensive care unit admission and mortality risk in patients with CAP [21–23].

However, despite their widespread use and proven benefits, the PSI and CURB-65 scoring systems are not without limitations. Due to their calculation complexity, these systems can be cumbersome, making them less practical in fast-paced clinical settings. Most importantly, these scoring models do not adequately account for patients’ inflammatory status, which is a crucial factor in the progression and prognosis of CAP. Given these limitations, there is an ongoing and vigorous search for new biomarkers that may more accurately reflect the inflammatory status and provide better prognostic information for patients with severe CAP. Researchers particularly focus on identifying biomarkers that can be easily and rapidly measured, thus seamlessly integrated into clinical practice.

The lactate/albumin ratio may have the potential to be a readily accessible biomarker for predicting mortality in CAP patients and overcoming the disadvantage of scoring systems. Numerous studies have demonstrated its association with mortality in critically ill patients, especially with sepsis [14, 15, 17, 24–26].

Recent studies have delved into the potential of LAR in predicting in-hospital mortality among patients with community-acquired pneumonia. In the study by Xu et al., the prognostic value of LAR in CAP patients admitted to the ICU was investigated and compared with many different biomarkers and the intensive care mortality predictor scores [SOFA, Simplified acute physiology score (SAPS II)] [18]. Age, mean arterial pressure, oxygen saturation, heart rate, SAPS II score, neutrophil-to-lymphocyte ratio, and LAR (> 1.6) were identified as independent risk factors for mortality. In the ROC analysis, the predictive ability of LAR for mortality was higher compared to other parameters and similar to the predictive abilities of SOFA and SAPS II. Erdogan et al. found that LAR (> 0.9) could be used as an independent indicator for mortality and correlated with Acute Physiology and Chronic Health Evaluation (APACHE II) and SOFA scores in patients hospitalized in the intensive care unit due to pneumonia and sepsis [15]. In a recent study, researchers investigated the predictive value of the LAR on myocardial injury in elderly patients with severe community-acquired pneumonia (SCAP) [27]. The study found that the area under the curve of LAR for predicting myocardial injury in elderly patients with SCAP was 0.737 (95% confidence interval 0.620–0.834). Furthermore, with a cut-off value of 1.21, the sensitivity and specificity of the prediction were 60.00% and 78.72%, respectively. In our study, the predictive power of the LAR was found to be moderate, consistent with previous research findings. However, our study identified a lower cut-off value compared to earlier studies. This discrepancy can be attributed to the inclusion of patients with varying pneumonia severity, both in the ICU and the ward. Consequently, we compared the LAR with scores that assess the severity of CAP rather than those predicting ICU mortality. Our findings demonstrated that the LAR was comparable to these commonly used severity scores. Furthermore, we found the sensitivity and specificity of the PSI and CURB-65 scores within the expected range of reliability in this patient population, which supports the reliability of our lactate/albumin results.

In our study, q-SOFA score was also evaluated and was shown to be associated with both ICU admission and mortality. Q-SOFA is a simple and rapid scoring system especially recommended for sepsis screening [28]. In a systematic review, Geladian et al. compared the CURB-65 and q SOFA in predicting pneumonia outcomes and they reported that the q-SOFA demonstrated a higher specificity of 86% and exhibited enhanced prognostic performance with an AUC of 0.747 in predicting ICU admissions [29]. Conversely, the CURB-65 scoring system displayed superior sensitivity at 76.52% and also achieved an AUC of 0.747 in the context of mortality prediction. Tokioka et al. examined the effectiveness of q-SOFA, CURB-65, and PSI in predicting in-hospital mortality and the need for ICU admission among hospitalized CAP patients [30]. They found that the prognostic performance of q-SOFA for in-hospital mortality and ICU admission was not significantly different from that of CURB-65 and PSI. In our study, we found that the AUC value for q-SOFA was 0.755 for ICU admission and 0.645 for predicting mortality. These values are slightly lower than those of CURB-65, PSI, and LAR. This indicates that although q-SOFA might not be the primary tool for predicting mortality, it remains a valuable practical resource for making quick decisions regarding the admission of pneumonia patients.

The study has a few limitations. It’s a retrospective study at a single centre, so the sample analysis was limited. Hence, the findings need to be interpreted with caution, particularly in various clinical settings. Second, LAR was calculated at a single time point; dynamic assessment could have better prognostic performance in predicting hospital mortality. Third, we couldn’t compare LAR with other prognostic biomarkers of pneumonia, like C-reactive or procalcitonin, due to missing data. Fourth, the number of non-survivors was relatively small (*n* = 27), which may have reduced the statistical power of comparisons between survivor and non-survivor groups.

## Conclusions

This study demonstrated that LAR can predict clinical deterioration in hospitalized patients with CAP who need to be admitted to the ICU and can be used as a biomarker to guide clinicians in the decision to admit patients to the intensive care unit.

## Electronic supplementary material

Below is the link to the electronic supplementary material.

Supplementary Material 1

## Data Availability

The research data used in this study are not publicly available due to ethical reasons but are available from the corresponding author upon reasonable request.

## Declarations

### Data sharing statement

The data associated with this research project is being made available to promote transparency and facilitate further scientific inquiry in the field. For inquiries regarding access to the data or any additional information, please contact the corresponding author.

## Abbreviations

CAP: Community-acquired pneumonia
ICU: Intensive care unit
CURB-65: Confusion, urea, respiratory rate, blood pressure, 65 years or older
PSI: Pneumonia severity index
SOFA: Sequential organ failure assessment
q-SOFA: Quick SOFA
LAR: Lactate to albumin ratio
ROC: Receiver operator curve
AUC: Area under the curve
SAPS II: Simplified acute physiology score
APACHE II: Acute Physiology and Chronic Health Evaluation
SCAP: Severe community-acquired pneumonia
