# Supplementary material for: Lactate to albumin ratio as a determinant of intensive care unit admission and mortality in hospitalized patients with community-acquired pneumonia
Source: BMC Pulm Med. 2025 May 9;25:224. doi: 10.1186/s12890-025-03698-7 (PMC12065318; doi:10.1186/s12890-025-03698-7)
Supplement: Supplementary file 1 — Supplementary Material 1 [file 12890_2025_3698_MOESM1_ESM.docx]

**Supplement Table 1. Pairwise Comparisons of ROC Curves Regarding the ICU Admission**

| PSI vs LAR | |
| --- | --- |
| Difference between areas | 0.0454 |
| Standard Error | 0.0574 |
| 95% Confidence Interval | -0.0672 - 0.158 |
| z statistic | 0.791 |
| p | 0.42 |
| CURB-65 vs LAR | |
| Difference between areas | 0.0759 |
| Standard Error | 0.0634 |
| 95% Confidence Interval | -0.0484 - 0.200 |
| z statistic | 1.198 |
| p | 0.23 |
| PSI vs CURB-65 | |
| Difference between areas | 0.0305 |
| Standard Error | 0.0326 |
| 95% Confidence Interval | -0.0333 - 0.094 |
| z statistic | 0.937 |
| p | 0.34 |
| PSI vs q-SOFA |  |
| Difference between areas | 0.0302 |
| Standard Error | 0.0528 |
| 95% Confidence Interval | -0.073-0.134 |
| z statistic | 0.573 |
| p | 0.56 |
| CURB-65 vs q-SOFA |  |
| Difference between areas | 0.0629 |
| Standard Error | 0.0557 |
| 95% Confidence Interval | -0.046-0.172 |
| z statistic | 1.129 |
| p | 0.25 |
| LAR vs q-SOFA |  |
| Difference between areas | 0.0141 |
| Standard Error | 0.0640 |
| 95% Confidence Interval | -0.111-0.140 |
| z statistic | 0.220 |
| p | 0.82 |

Definition of Abbreviations: PSI: Pneumonia severity index. LAR: Lactate–albumin ratio. CURB-65: Confusion, blood urea nitrogen, respiratory rate, blood pressure, age >65. qSOFA: Quick sequential organ failure assessment.

**Supplement Table 2. Pairwise Comparisons of ROC Curves Regarding the ICU Admission**

| PSI vs LAR | |
| --- | --- |
| Difference between areas | 0.0390 |
| Standard Error | 0.0665 |
| 95% Confidence Interval | -0.0914 - 0.169 |
| z statistic | 0.586 |
| p | 0.55 |
| CURB-65 vs LAR | |
| Difference between areas | 0.0180 |
| Standard Error ^a^ | 0.0739 |
| 95% Confidence Interval | -0.127 - 0.163 |
| z statistic | 0.243 |
| p | 0.80 |
| PSI vs CURB-65 | |
| Difference between areas | 0.0210 |
| Standard Error | 0.0448 |
| 95% Confidence Interval | -0.0667 - 0.109 |
| z statistic | 0.469 |
| p | 0.63 |
| PSI vs q-SOFA |  |
| Difference between areas | 0.0647 |
| Standard Error | 0.0636 |
| 95% Confidence Interval | -0.059-0.189 |
| z statistic | 1.018 |
| p | 0.30 |
| CURB-65 vs q-SOFA |  |
| Difference between areas | 0.0860 |
| Standard Error | 0.0697 |
| 95% Confidence Interval | -0.091-0.173 |
| z statistic | 0.608 |
| p | 0.54 |
| LAR vs q-SOFA |  |
| Difference between areas | 0.106 |
| Standard Error | 0.0845 |
| 95% Confidence Interval | -0.059-0.271 |
| z statistic | 1.251 |
| p | 0.21 |

Definition of Abbreviations: PSI: Pneumonia severity index. LAR: Lactate–albumin ratio. CURB-65: Confusion, blood urea nitrogen, respiratory rate, blood pressure, age >65. q-SOFA: Quick sequential organ failure assessment.
